# Clinical Evaluation of a Defined Zeolite-Clinoptilolite Supplementation Effect on the Selected Blood Parameters of Patients

**DOI:** 10.3389/fmed.2022.851782

**Published:** 2022-05-27

**Authors:** Sandra Kraljević Pavelić, Lara Saftić Martinović, Jasmina Simović Medica, Marta Žuvić, Željko Perdija, Dalibor Krpan, Sandra Eisenwagen, Tatjana Orct, Krešimir Pavelić

**Affiliations:** ^1^Faculty of Health Studies, University of Rijeka, Rijeka, Croatia; ^2^Department of Biotechnology, University of Rijeka, Rijeka, Croatia; ^3^General Hospital Pula, Pula, Croatia; ^4^Medical Centre CIIM Plus, Maribor, Slovenia; ^5^Polyclinic “K—Center” for Internal Medicine, Gynaecology, Radiology, Physical Medicine and Rehabilitation, Zagreb, Croatia; ^6^Panaceo International GmbH, Villach, Austria; ^7^Institute for Medical Research and Occupational Health, Zagreb, Croatia; ^8^Faculty of Medicine, Juraj Dobrila University of Pula, Pula, Croatia

**Keywords:** PMA-zeolite, clinoptilolite, metals, minerals, clinical study, safety

## Abstract

The natural clinoptilolite material is an inorganic crystal mineral called zeolite. It has been extensively studied and used in industrial applications and veterinary and human medicine due to positive effects on health. Limited data is available in the scientific literature about its effects on the levels of physiologically relevant minerals in the human organism. Accordingly, we performed a comprehensive and controlled monitoring of the relevant mineral and contaminants levels in human subjects supplemented with a certified clinoptilolite material within three clinical trials with different supplementation regimens. Effects of a registered and certified clinoptilolite material PMA-zeolite on selected mineral and metal levels were determined by standard biochemical methods and inductively coupled plasma mass spectrometry (ICP-MS) in the blood of subjects enrolled in three clinical trials: short-term (28 days, Mineral Metabolism and selected Blood Parameters study MMBP), medium-term (12 weeks, Morbus Crohn study), and long-term (4 years, Osteoporosis TOP study) supplementation. Lower concentrations were observed for copper (Cu) in patients with osteoporosis, which normalized again in the long-term supplementation trial, whereas sodium (Na) and calcium (Ca) levels diminished below the reference values in patients with osteoporosis. In the short- and long-term supplementation trials, increased levels of lead (Pb) were observed in PMA-zeolite-supplemented subjects, which decreased in the continued long-term supplementation trial. Increased levels of aluminum (Al) or Pb attributable to eventual leakage from the material into the bloodstream were not detected 1 h after intake in the short-term supplementation trial. Nickel (Ni) and Al were statistically significantly decreased upon long-term 4-year supplementation within the long-term supplementation trial, and arsenic (As) was statistically significantly decreased upon 12-weeks supplementation in the medium-term trial. Alterations in the measured levels for Na and Ca, as well as for Pb, in the long-term trial are probably attributable to the bone remodeling process. Checking the balance of the minerals Cu, Ca, and Na after 1 year of supplementation might be prescribed for PMA-supplemented patients with osteoporosis.

**Clinical Trial Registration:**

[https://clinicaltrials.gov], identifiers [NCT03901989, NCT05178719, NCT04370535, NCT04607018].

## Introduction

The veterinary applications of natural zeolite-clinoptilolite have been documented in the scientific literature for more than a decade. Indeed, clinoptilolite materials, such as for example PMA-zeolite, showed a number of positive effects on health, including antioxidative, immunostimulatory effects, antidiarrheal effects, positive effects on the bones, and anti-tumor effects but its usage in humans has not been comprehensively evaluated in detail within large controlled clinical studies (1, 2). Accordingly, data on its documented systemic effects on the human body are limited. Toxicology studies of this material are scarcely covered in the scientific literature. A tribomechanically activated clinoptilolite material has for example, been studied previously and proved as generally safe by Pavelic et al. (3, 4). Moreover, the systemic effects of natural zeolite-clinoptilolite are not well-understood, as its mechanism of action substantially differs from organic molecules, such as those in the pharmaceutical products or biotechnology-based medical products. This material is indeed a volcanic tuff, which is characterized by high thermal stability and resistance to chemical substances, and the *in vivo* applications rely on its physical-chemical properties (5). Clinoptilolite contains alkali and alkaline earth cations within its crystal framework, and some of them [mainly calcium (Ca) or sodium (Na)] can be easily exchanged in the environment through the ion-exchange process. This process may occur in exchange with contaminants, such as lead (Pb), nickel (Ni), cadmium (Cd), or arsenic (As), due to their affinity toward the crystal framework of zeolite. This property was exploited not only in several industrial applications but also for detoxification effects *in vivo*, as presented previously in the scientific literature (6–11). Due to this well-documented ion-exchange property, questions arose on the potential effect of clinoptilolite on physiologically relevant minerals in the *in vivo* applications. With this rationale, we collected relevant data on levels of minerals and metals in the blood of subjects enrolled in different clinical trials and supplemented with a registered and certified clinoptilolite material, namely the PMA-zeolite material. Short-, medium-, and long-term clinical trials were performed to monitor the effects of PMA-zeolite in patients with osteoporosis supplemented with PMA-zeolite for 4 years (long-term; ClinicalTrials.gov identifier: NCT03901989), and (NCT05178719)^1^ Crohn patients supplemented with PMA-zeolite for 12 weeks (medium-term; ClinicalTrials.gov identifier: NCT04370535), and healthy volunteers supplemented with PMA-zeolite for 28 days (short-term; ClinicalTrials.gov identifier: NCT04607018). Previously, PMA-zeolite was also tested in a clinical trial on athletes, where it did not show any negative side effects or impacts on the measured blood parameters, including minerals (12). Herein, the main goal of the presented data from clinical trials was to monitor the general safety of the medical device PMA-zeolite in human subjects with relevance to the blood levels of minerals and metals within short-, medium-, and long-term supplementation regimens.

## Materials and Methods

### Clinoptilolite PMA-Zeolite Material

The clinoptilolite material PMA-zeolite was provided by Panaceo International GmbH, Austria. PMA (Panaceo Micro Activation)-zeolite (patent WO2018/100178A1) is a certified European medical device subjected to required toxicology tests performed according to the OECD and ISO guidelines. The dosage was adjusted according to the certification of the PMA-zeolite material and EFSA safety data on clinoptilolite materials (13). The treatment period was adjusted according the pathology study and in agreement with medical doctors leading the trials. The explorative trial on healthy subject (MMBP) was designed as a preliminary safety study and accordingly, it lasted 28 days.

### Clinical Trials

#### Explorative Clinical Trial on Healthy Subjects (Mineral Metabolism and Selected Blood Parameters)

The clinical trial on healthy subjects (Mineral Metabolism and selected Blood Parameters, MMBP) (NCT04607018) took place in Croatia, under supervision of the University of Rijeka at the General Hospital Pula, Croatia. The short-term effect of PMA-zeolite was evaluated on minerals and selected blood parameters of subjects supplemented with PMA-zeolite for 28 days. Two groups of volunteers were involved in the study, comprising a total of 15 subjects, namely the CHRONIC group and NAÏVE group (Supplementary Tables 1, 2). The NAÏVE group consisted of new PMA-zeolite users that began to take PMA-zeolite for the first time at a dosage of 6 g/day for 28 days at the beginning of the trial. A total of seven healthy volunteers were in the NAÏVE group, with a mean age of 45 years among male and female participants. The CHRONIC group (control) consisted of eight subjects; all of them were frequent daily users of PMA-zeolite, who already took PMA-zeolite at a dosage of 6 g or more per day for 6 months and up to 8 years before the start of the trial (Supplementary Material). The subjects enrolled in the CHRONIC group were also healthy males and females, with a mean age of 56 years. *Inclusion criteria for all groups*: Healthy volunteers at least 18 years old who provided informed consent. *Exclusion criteria (only for the* NAÏVE *group*): Chronic disease (cancer, renal disease, neurodegenerative disorders, metabolic disorders, or diabetes), recent vaccinations, pregnancy or breastfeeding, and food supplements. Additional information on involved subjects is given in the Supplementary Material along with standard blood parameters reference values (Supplementary Table 3). The determined blood parameters (Table 1) were measured in the NAÏVE and CHRONIC groups at the beginning of the study (time point T0), 1 h after intake of PMA-zeolite (only the NAÏVE group, time point T1), and after 28 days (time point T2). Contaminants for the CHRONIC group were measured at one-time point only (T2). The study was conducted according to the guidelines of the Declaration of Helsinki for Research on Human Subjects 1989 and was approved by the Ethical Review Committee of the General Hospital Pula, Croatia (17 December 2015, register number: 10095/15.1). All subjects provided written informed consent prior to participating in this investigation. The sample size of around 15 subjects is also used in the literature for exploratory clinical trials (14).

**TABLE 1:** Blood parameters evaluated in the Mineral Metabolism and selected Blood Parameters (MMBP) study, Osteoporosis “Treatment of osteoporosis by Panaceo” (TOP) study, and Morbus Crohn study.

Clinical trial	Measured standard blood parameters
**MMBP study (NCT04607018)**	
Sample size: 15 volunteers (7 Naïve PMA users and 8 chronic PMA users)	Na, K, Zn, Fe, Mg, and Ca mineral levels; AST; ALT; GGT; CREAT; GF evaluation; and contaminants panel measured by ICP-MS
**Osteoporosis TOP study (NCT03901989)**	
Enrolled patients: 100 First study year comprised 50 patients in the placebo group and 50 patients in the verum group (PMA supplementation)	CREAT, ALP, P, Ca, mineral and contaminants panel measured by ICP-MS
**Morbus Crohn study (NCT04370535)**	
Enrolled patients: 30 comprising patients with uncontrolled Crohn disease + placebo (15 patients) and patients with uncontrolled Crohn disease + PMA (15 patients) Healthy volunteers: 10 control group + placebo (five test persons) and control group + PMA (five test persons)	Sedimentation; blood count; Na, K, Cl, Fe, Mg, and Ca mineral levels; urea; CREAT; AST; ALT; GGT; bilirubin; CRP; procalcitonin; ferritin; calprotectin; and contaminants panel measured by ICP-MS

*The sample size of each study is also presented (additional information are given in the Supplementary Material).*

*AST, Aspartate-aminotransferase; ALT, Alanine-aminotransferase; GGT, Gamma-glutamyltransferase; CREAT, Creatinine; ALP, Alkaline phosphatase; GF, Glomerular filtration; CRP, C-reactive protein.*

#### Osteoporosis Study (Treatment of Osteoporosis by Panaceo)

The clinical results of the first year of the TOP study (TOP1) are published in Kraljević Pavelić et al. (15). Herein, we report on the concentrations of minerals and contaminants in the blood of patients enrolled within this randomized, placebo-controlled, double-blinded study (NCT03901989) aimed to measure the long-term effects of PMA-zeolite on the bones of patients with osteoporosis who were supplemented with PMA-zeolite for a period up to 3 or 4 years (36 or 48 months, respectively, TOP1—first year of TOP study, TOP2—second year of the TOP study, TOP3—third year of the TOP study, and TOP4—fourth year of the TOP study). The initial number of PMA-zeolite-supplemented subjects in the first year was 50. The placebo group in the first year of the study (*n* = 50) received microcrystalline cellulose powder similar in appearance to PMA-zeolite, as already used in previous studies (12, 16). All placebo subjects were given PMA-zeolite (Verum group, PMA-supplemented subjects) after the end of TOP1 and for further study duration. After the fourth year, the total number of evaluated test subjects supplemented with PMA-zeolite was 62. The PMA-zeolite-treated patients received 9 g/day of PMA-zeolite for 36 months (patients enrolled within the first year as placebo) or 48 months (Verum group supplemented with PMA-zeolite from the beginning of the study). *Inclusion criteria*: One hundred male and female patients with osteoporosis (Croatian Caucasians) who were 56–74 years of age, BMD T-score of 2.5 or lower, untreated or subjects with failed osteoporosis treatment so far. *Exclusion criteria*: Other severe diseases such as cancer, autoimmune disease, chronic renal failure, and secondary osteoporosis. Female subjects who were pregnant were also excluded.

#### Morbus Crohn Clinical Trial

This pilot study (NCT04370535) was a randomized, placebo-controlled, double-blinded study comprising subjects suffering from Morbus Crohn. The four subject groups involved in the study are presented in Table 1. The details of the study are presented in the Supplementary Material.

*Inclusion criteria of healthy volunteers*: Absence of gastroenterological symptoms and any regular medication prescription assessed by anamnesis. *Inclusion criteria for patients*: Patients with confirmed Crohn’s disease treated with standard therapy without appropriate disease remission. *Exclusion criteria:* Signs of acute bacterial infection (fever > 38°C, nausea, and/or vomiting), other chronic disease (cancer, renal disease, neurodegenerative disorders, metabolic disorders, or diabetes), pregnancy or breastfeeding. The subjects received 6 g/day of PMA-zeolite for 12 weeks. The parameters were measured at the beginning and at the end of the study.

### Analyses of Minerals and Contaminants in the Blood

Standard biochemical laboratory blood analyses and part of the mineral measurements in the MMBP study were performed in corresponding accredited biochemical clinical laboratories by use of standard methods in place for all subjects involved in the clinical trials at the beginning and at the end of the studies. The measured parameters are given in Table 1. Elemental measurements in the blood of enrolled subjects were performed by use of inductively coupled plasma mass spectrometry (ICP-MS) analysis as described in Živković et al. (17). Briefly, measurement of metal levels (Pb, Cd, Hg, and As in blood and Al and Ni in serum) and mineral levels (Se, Cu, Zn, Fe, Ca, Na, Mg, and K in serum) were carried out by using ICP-MS (Agilent 7500cx, Agilent Technologies, Japan). Haemolysed samples were not included in the analysis. Calibration and internal standards (Ge, Rh, Tb, Ir, and Lu) were prepared from single element standards (SCP Science, Quebec, Canada). Before analysis, blood and serum samples were diluted (1:70 and 1:20, respectively) in a solution containing 0.7 mM ammonia, 0.01 mM EDTA, 0.07% Triton X-100, and 3 μg/L of internal standard in ultrapure water (GenPure System, TKA, DE). Each sample was prepared and analyzed in duplicate. Blanks, reference materials, and calibration standards were prepared in the same way as the samples and analyzed accordingly. The calibration and instrument sensitivity control were performed by re-analyzing selected calibration standards every 30 analyses. Results for chromium (Cr) measurements are not presented in this paper due to pre-analytical and analytical challenges in Cr detection. The reference values used for interpretation of measurements in each study are given in Table 2.

**TABLE 2:** Referent values used for interpretation of levels of minerals or contaminants measured in the blood of subjects involved in presented clinical studies.

Study	Referent values
MMBP study blood electrolytes reference values evaluated by standard biochemical methods.	**Na:** 137–146 mmol/L
	**K:** 3.9–5.1 mmol/L
	**Zn:** M: 11.1–19.5 μmol/L, F: 10.7–17.5 μmol/L
	**Fe:** M: 11–32 μmol/L, F: 8–30 μmol/L
	**Mg:** 0.65–1.05 mmol/L
	**Ca:** 2.14–2.53 mmol/L
Osteoporosis TOP study and Morbus Crohn study. The mineral panel was evaluated by ICP-MS. Mayo Clinic Laboratory standard reference values for mineral levels in blood as set on May 2017.	**Mg:** 17–23 mg/L
	**Ca:** 89–101 mg/L
	**Na:** 3100–3350 mg/L
	**K:** 140–200 mg/L
	**Fe** M: 550–1600 μg/L F: 400–1550 μg/L
	**Zn:** 660–1100 μg/L
	**Cu:** 750–1450 μg/L
	**Se:** 70–150 μg/L
Contaminants (all studies). The contaminant panel was evaluated by ICP-MS. Mayo Clinic Laboratory standard reference values for contaminants levels in blood as set on May 2017.	**Pb** ≤ 50.0 μg/L
	**Hg** 0–2 μg/L
	**Cd** < 1 μg/L
	**As** < 12 μg/L
	**Al** < 8 μg/L
	**Ni** < 2 μg/L

### Statistical Analyses and Sample Size

The collected data were statistically evaluated using the data analysis software system Statistica, version 12, from Dell Inc. (2015). Categorical variables are presented with frequencies or percentages and comparisons done using the Pearson chi-square test or Fisher exact test, where appropriate. Normally distributed continuous variables (distribution tested with the Kolmogorov–Smirnov test) are presented as means with standard deviations or medians with interquartile ranges (IQRs), where appropriate. Comparisons of variables in two groups were done using the parametric *t*-test and the non-parametric Mann Whitney *U* test, where appropriate, and pairwise comparisons using the paired *t*-test or Wilcoxon matched pairs test, where appropriate. Multifactor comparisons (between groups and subgroups or between study time points and groups) were done using factorial ANOVA or repeated measures ANOVA within-factors, where appropriate. Predictors of overall health condition at the end of the study were analyzed applying multiple logistic regression modeling. Statistical significance was determined at the level of 0.05.

## Results

### Mineral Metabolism and Selected Blood Parameters Study

In the short-term PMA-zeolite supplementation study, no alterations in concentrations of physiologically relevant minerals measured by standard laboratory tests (Fe, Na, K, Ca, Mg, and Zn) were observed in the PMA-zeolite long-term supplemented CHRONIC group (Table 3). In the NAÏVE group, starting values (T0) for Fe were under referent values for one subject and increased upon PMA-zeolite supplementation (T2). Subjects in the NAÏVE group with increased Zn values at the beginning of the study (T0) had lower or normalized Zn concentrations upon PMA-zeolite supplementation (T2). Statistically significantly higher Ca values were observed in the NAÏVE group in comparison with those in the CHRONIC group (*p* = 0.001). Oppositely, significantly higher Mg (*p* = 0.002) and Na (*p* = 0.028) levels were observed in the CHRONIC group in comparison with those in the NAÏVE group. However, Ca, Mg, and Na, as well as K, values in both groups were in the normal range, and these results seem to have no clinical relevance (Table 3). No alterations in the concentrations of physiologically relevant minerals (i.e., Fe, Na, K, Ca, Mg, and Zn) were observed in the CHRONIC group.

**TABLE 3:** Results of the mineral analyses in serum of the subjects enrolled in the MMBP study (NCT04607018), sampled at the beginning of the study (T0) and after 28 days of PMA-zeolite supplementation (T2).

Group	Time point	Subject	Gender	Fe	Na	K	Ca	Mg	Zn
									
				(μmol/L)	(mmol/L)	(mmol/L)	(μmol/L)	(μmol/L)	(μmol/L)
**NAÏVE**	**T0**	1	M	26.6	138	4.6	2.5	0.9	18.6
	**T2**			31.0	138	4.5	2.4	0.8	12.1
	**T0**	2	M	19.1	141	4.1	2.5	0.8	22.7*
	**T2**			29.7	140	4.1	2.5	0.8	17.5*#
	**T0**	3	F	4.0*	141	5.1	2.5	0.9	17.9
	**T2**			**6.1#**	140	4.4	2.3	0.9	**14.8#**
	**T0**	4	M	16.8	139	4.5	2.4	0.8	14.8
	**T2**			23.1	140	4.9	2.4	0.7	13.8
	**T0**	5	F	8.5	138	4.4	2.4	0.9	15.2
	**T2**			11.6	139	4.5	2.3	0.8	12.7
	**T0**	6	F	17.8	141	4.2	2.5	0.8	NA
	**T2**			14.2	141	4.4	2.5	0.9	**13.4#**
	**T0**	7	M	16.5	143	4.7	2.6*	0.8	NA
	**T2**			12.5	144	4.6	**2.5#**	0.8	**14.1#**
**CHRONIC**	**T0**	1	F	18.7	142	3.8	2.4	0.9	15.8
	**T2**			18.4	145	4.2	2.5	0.9	NA^¥^
	**T0**	2	F	21.8	140	4.4	2.4	0.9	14.7
	**T2**			19.4	143	4.2	2.4	1.0	NA
	**T0**	3	F	12.8	142	4.5	2.4	0.8	14.0
	**T2**			14.2	141	4.8	2.3	0.9	NA
	**T0**	4	M	20.8	141	4.5	2.4	0.9	14.4
	**T2**			19.7	145	4.6	2.5	1.0	NA
	**T0**	5	F	21.3	140	4.4	2.3	0.9	15.6
	**T2**			13.8	140	5.2	2.4	0.9	NA
	**T0**	6	M	24.7	141	4.8	2.4	0.8	16.8
	**T2**			18.6	144	4.9	2.5	0.9	NA
	**T0**	7	F	13.6	144	4.4	2.4	0.9	15.5
	**T2**			15.1	142	4.8	2.5	1.0	NA
	**T0**	8	F	13.7	140	4.3	2.4	0.8	13.2
	**T2**			15.3	141	4.4	2.4	0.8	NA

*Values exceeding referent parameters in bold.

*#Parameters that improved toward referent values upon PMA-zeolite supplementation denoted in bold.*

*^¥^NA–some measurements of Zn in the chronic group were not presented due to quality problems with reagents assessed upon completion of the study.*

The blood parameters tested for subjects in the CHRONIC group were all in the normal range, except for a slight variation in the first measurement of CREAT for one subject and the second measurement of the glomerular filtration value for two subjects (Supplementary Table 4). However, these findings were assumed not to be clinically relevant in the context of the general health condition of the subject. Glomerular filtration values were critical for subject 7 in the CHRONIC group, which is in line with the age (90 Y) and chronic health status (cardiac insufficiency). The NAÏVE group presented with generally lower glomerular filtration values in comparison with those in the CHRONIC group, where the difference among female subjects was statistically significant (*p* = 0.048) (Supplementary Table 4). These values were ameliorated upon PMA-zeolite supplementation in three subjects. Also, three subjects in the NAÏVE group had slightly altered ALT values that normalized upon PMA-zeolite supplementation. CREAT values were increased in one subject in the NAÏVE group only at the beginning of the study, but these values decreased substantially upon PMA-zeolite supplementation (Supplementary Table 4).

Some metal levels in certain subjects within the NAÏVE group at the beginning of the study (T0) exceeded referent values but showed statistically relevant decreases upon PMA-zeolite supplementation (Hg *p* = 0.003, Cd *p* = 0.040) (Table 4). Elevated levels of cadmium (Cd) that were observed in three subjects within the NAÏVE group at the beginning of the study can be attributed to smoking habits. In all these subjects, Cd levels decreased upon PMA-zeolite supplementation. Moreover, levels of mercury (Hg) and arsenic (As) were increased above referent values in some subjects both in the NAÏVE and CHRONIC groups. Lead (Pb) values were slightly increased upon PMA-zeolite supplementation in the NAÏVE group but still in the referent range. Pb values were also significantly higher in the CHRONIC group in comparison to those in the NAÏVE group (*p* = 0.040). One subject in the NAÏVE group had elevated Ni values that were not reduced upon PMA-zeolite supplementation. All values for Al in the CHRONIC and NAÏVE groups were below referent values.

**TABLE 4:** Metal concentrations in tested subjects enrolled in the MMBP study (NCT04607018) by ICP-MS analysis.

Group	Subject	Time point	Age	Gender	Pb blood	Hg blood	Cd blood	As blood	Al serum	Ni serum
										
					(μg/L)	(μg/L)	(μg/L)	(μg/L)	(μg/L)	(μg/L)
**NAÏVE**	1	T0	54	M	14.2 ± 0.2	28.3 ± 0.2*	0.1 ± 0.0	16.7 ± 0.1*	5.5 ± 2.1	1.0 ± 0.0
		T1			14.3 ± 0.1	29.3 ± 0.0*	0.2 ± 0.0	17.5 ± 0.3*	5.9 ± 0.5	1.1 ± 0.0
		T2			18.6 ± 0.1	21.4 ± 0.4*#	0.2 ± 0.1	**6.1 ± 0.1#**	6.5 ± 0.0	1.0 ± 0.0
	2	T0	35	M	33.0 ± 0.0	1.3 ± 0.1	8.5 ± 0.3*	2.7 ± 0.1	6.2 ± 0.3	4.5 ± 0.2*
		T1			36.0 ± 0.9	1.3 ± 0.0	9.1 ± 0.1*	2.6 ± 0.2	8.1 ± 0.0	5.4 ± 0.1*
		T2			30.5 ± 0.1	1.0 ± 0.1	7.8 ± 0.1*#	1.3 ± 0.1	8.7 ± 3.7	5.8 ± *0.6
	3	T0	48	F	8.5 ± 0.2	4.5 ± 0.1	0.4 ± 0.0	2.2 ± 0.1	8.8 ± 4.9	0.9 ± 0.1
		T1			8.3 ± 0.3	4.3 ± 0.1	0.4 ± 0.0	2.0 ± 0.1	5.4 ± 0.8	0.8 ± 0.0
		T2			13.3 ± 0.0	4.1 ± 0.1	0.4 ± 0.0	2.1 ± 0.1	7.2 ± 0.0	0.9 ± 0.1
	4	T0	40	M	22.8 ±	15.7 ± 2.0*	0.5 ± 0.0	12.1 ± 0.2	8.4 ± 1.6	0.8 ± 0.2
		T1			17.8 ± 0.3	13.3 ± 0.2*	0.3 ± 0.0	10.9 ± 0.1	7.2 ± 0.3	0.7 ± 0.0
		T2			26.8 ± 0.3	12.3 ± 0.0*#	0.2 ± 0.0	16.3 ± 0.3*	6.3 ± 0.4	0.7 ± 0.0
	5	T0	45	F	9.7 ± 0.0	4.2 ± 0.1	0.5 ± 0.0	6.9 ± 0.4	6.4 ± 0.9	0.8 ± 0.1
		T1			9.6 ± 0.1	4.4 ± 0.0	0.5 ± 0.0	6.6 ± 0.2	9.8 ± 4.5	0.7 ± 0.1
		T2			31.0 ± 0.2	4.1 ± 0.0	0.4 ± 0.0	3.5 ± 0.0	6.7 ± 0.0	0.9 ± 0.3
	6	T0	48	F	11.5 ± 0.1	3.1 ± 0.1	2.3 ± 0.0*	3.1 ± 0.3	4.0 ± 0.0	0.9 ± 0.4
		T1			11.6 ± 0.1	3.1 ± 0.1	2.3 ± 0.0*	3.1 ± 0.1	6.1 ± 3.3	0.8 ± 0.1
		T2			15.0 ± 0.1	2.3 ± 0.1	1.8 ± 0.1*#	2.5 ± 0.0	5.4 ± 1.3	0.8 ± 0.1
	7	T0	46	M	23.1 ± 0.3	15.9 ± 0.0*	2.6 ± 0.0*	18.6 ± 0.4*	7.6 ± 2.2	0.9 ± 0.1
		T1			22.7 ± 0.6	15.6 ± 0.1*	2.6 ± 0.1*	17.9 ± 0.0*	5.0 ± 0.0	0.9 ± 0.1
		T2			24.1 ± 0.4	10.6 ± 0.1*#	2.3 ± 0.0*#	**3.7 ± 0.1#**	5.2 ± 0.4	0.9 ± 0.0
**CHRONIC**	1	T2	62	F	28.7 ± 0.1	9.6 ± 0.0	0.5 ± 0.0	11.5 ± 0.2	4.6 ± 0.5	1.0 ± 0.0
	2	T2	36	F	22.9 ± 0.1	5.4 ± 0.2	0.8 ± 0.0	3.0 ± 0.2	7.1 ± 4.5	0.9 ± 0.1
	3	T2	42	F	62.3 ± 0.8*	18.8 ± 0.2*	0.4 ± 0.0	27.4 ± 0.5*	5.3 ± 0.5	1.0 ± 0.0
	4	T2	40	M	36.4 ± 0.2	6.4 ± 0.0	0.9 ± 0.0	3.2 ± 0.0	6.0 ± 0.5	1.5 ± 0.0
	5	T2	63	F	35.2 ± 0.5	9.0 ± 0.0	0.4 ± 0.0	13.1 ± 0.5*	6.1 ± 2.2	0.8 ± 0.0
	6	T2	68	M	43.9 ± 0.7	10.4 ± 0.1*	0.2 ± 0.0	16.8 ± 0.0*	3.7 ± 0.2	1.0 ± 0.1
	7	T2	90	F	44.7 ± 0.1	1.1 ± 0.1	0.5 ± 0.0	1.4 ± 0.1	4.4 ± 0.2	0.8 ± 0.0
	8	T2	48	F	19.7 ± 1.4	2.5 ± 0.0	0.6 ± 0.0	17.0 ± 0.5*	6.4 ± 1.3	1.0 ± 0.1

*T0, beginning of the study; T1, values 1 h after PMA-zeolite oral intake in the NAÏVE group; T2, end of study after 28 days of PMA-zeolite supplementation. Results are presented as mean concentration ± standard deviation (SD) of duplicated measurements of samples.*

*Values exceeding referent parameters denoted in bold.

***#**Parameters that improved toward referent values upon PMA-zeolite supplementation denoted in bold.*

In summary, PMA-zeolite supplementation did not alter mineral levels and did not confer to eventual increase of metal contaminants in the blood of healthy volunteers (NAÏVE group) after 28-days supplementation.

### Osteoporosis Study, TOP Study

**FIGURE 1:**
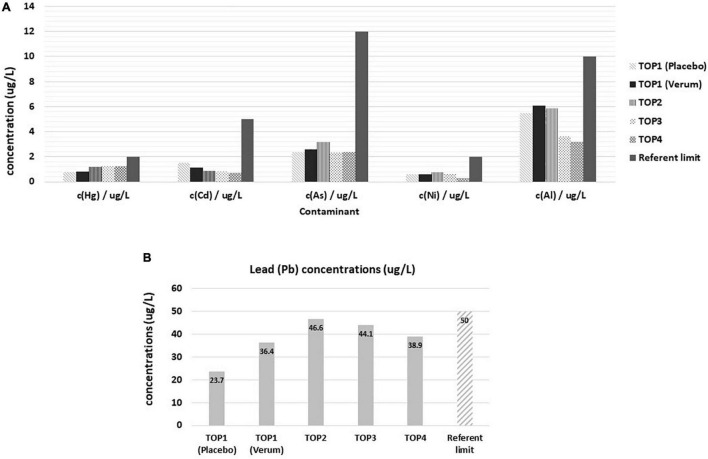
Levels of contaminants in Placebo and Verum groups during the osteoporosis TOP study course of 4 years with corresponding reference limits. **(A)** Concentrations of mercury, cadmium, arsenic, nickel, and aluminum. Statistically lower values were observed in TOP4 in comparison with TOP3 for nickel (*post-hoc* analysis, *p* < 0.001). **(B)** Concentrations of lead in the osteoporosis TOP study course of 4 years. Statistically lower levels of lead were observed in TOP4 comparison with TOP3 (*post-hoc* analysis, *p* < 0.001). TOP1-Placebo (without PMA-zeolite supplementation); TOP1-Verum, end of the first year of PMA-zeolite supplementation; TOP2-Verum 2Y, end of the second year of PMA-zeolite supplementation; TOP3-Verum 3Y, end of the third year of PMA-zeolite supplementation; TOP4-Verum 4Y, end of the fourth year of PMA-zeolite supplementation.

Standard blood measurements in all PMA-zeolite-treated subjects during the clinical trial were in the reference range (data not shown). The majority of mineral levels were also in the referent range, even though statistically relevant fluctuations in PMA-zeolite-treated patients in comparison with placebo were transiently observed (Supplementary Tables 9–13). Statistically significant changes in mineral levels in subjects treated with PMA-zeolite that were below the referent values, thus having potential clinical relevance, were observed, including Cu and Na (Table 5 and Supplementary Tables 9–13). Metal concentrations were all within the reference value ranges (Figure 1A). Concentrations of metals (mean values of all Verum groups) were tested against the referent value (upper limit) and the results showed the measured values were significantly lower in all cases (*p* < 0.001). Statistically higher levels in comparison with placebo were observed for Pb (*p* < 0.001) in the Verum groups (Table 5 and Figure 1B). In the fourth year, however, a statistically relevant decrease of Pb levels was observed in the PMA-zeolite-treated group in comparison with the PMA-zeolite-treated group in the third year (Supplementary Table 11). On the contrary, aluminum (Al) and nickel (Ni) levels were statistically significantly lower in PMA-zeolite-treated patients after four years of supplementation (*p* < 0.001) (Table 5). Previously, the Al detoxification properties of clinoptilolite materials, including PMA-zeolite, were shown *in vivo* on rats as well (8).

**TABLE 5:** Concentrations of selected metals measured in the TOP study in Placebo, Verum 1Y (time point 1), Verum 2Y (time point 2), Verum 3Y (time point 3), and Verum 4Y (time point 4) in comparison with each other.

	*N*	Placebo	*N*	Verum time point 1	*N*	Verum time point 2	*N*	Verum time point 3	*N*	Verum time point 4	*p#*	Reference values (μg/L)
Pb blood (μg/L)	29	23.7 ± 11.2	29	36.4 ± 15.7	68	45.3 ± 17.3	56	44.1 ± 15.8	57	38.9 ± 15.7	<0.001*	≤50.0
Hg blood (μg/L)	29	0.8 ± 0.8	29	0.8 ± 0.8	69	1.2 ± 1.4	56	1.2 ± 1.4	58	1.3 ± 1.6	0.426	0–2
Cd blood (μg/L)	29	1.5 ± 1.2	30	0.8 ± 0.8	69	0.8 ± 1.0	56	0.8 ± 1.0	58	0.7 ± 1.1	0.051	<5
As blood (μg/L)	29	2.4 ± 1.3	30	2.8 ± 2.1	69	3.3 ± 3.8	56	2.2 ± 2.5	58	2.6 ± 4.5	0.277	<12
Ni serum (μg/L)	30	0.6 ± 0.2	30	0.7 ± 0.2	69	0.8 ± 0.2	56	0.6 ± 0.3	58	0.3 ± 0.1	<0.001*	<2
Al serum (μg/L)	30	5.5 ± 1.1	30	6.7 ± 1.9	69	5.9 ± 2.2	56	3.6 ± 1.4	58	3.3 ± 1.0	<0.001*	<10
Mg serum (mg/L)	30	19.3 ± 1. 4	30	18.5 ± 1.8	69	18.3 ± 1.8	56	19.6 ± 1.7	58	20.5 ± 1.9	<0.001*	17–23
Ca serum (mg/L)	30	91.3 ± 4.2	30	90.6 ± 3.2	68	89.6 ± 3.5	56	88.7 ± 5.2	58	83.3 ± 5.4	<0.001*	89–101
Se serum (μg/L)	30	92.3 ± 17.5	30	89.3 ± 15.8	68	98.0 ± 16.4	56	83.1 ± 14.3	58	86.0 ± 17.9	<0.001*	70–150
Fe serum (μg/L)	30	998.4 ± 247.8	27	1034.7 ± 248.6	67	1016.9 ± 496.9	55	892.9 ± 278.3	58	918.9 ± 259.3	0.115	550–1600
Cu serum (μg/L)	30	1134.4 ± 167.0	27	1081.0 ± 119.6	68	942.5 ± 224.0	56	762.3 ± 272.9	58	949.3 ± 173.2	<0.001*	750–1450
Zn serum (μg/L)	30	702.5 ± 96.6	27	708.6 ± 92.8	67	667.1 ± 96.0	56	688.1 ± 97.3	58	723.9 ± 93.5	0.016*	660–1100
Na serum (mg/L)	30	3140.1 ± 85.6	29	3113.3 ± 117.2	67	2945.9 ± 153.1	56	3011.6 ± 120.5	58	2936.5 ± 169.4	0.001*	3100–3350
K serum (mg/L)	30	166.0 ± 14.5	29	158.7 ± 17.9	66	154.5 ± 18.1	56	154.5 ± 14.8	58	146.9 ± 16.2	0.001*	140–200

*Presented are mean values ± standard deviations (SDs).*

*Statistically relevant differences at p < 0.05, ANOVA repeated measures test.

*#Comparison of values between groups.*

**FIGURE 2:**
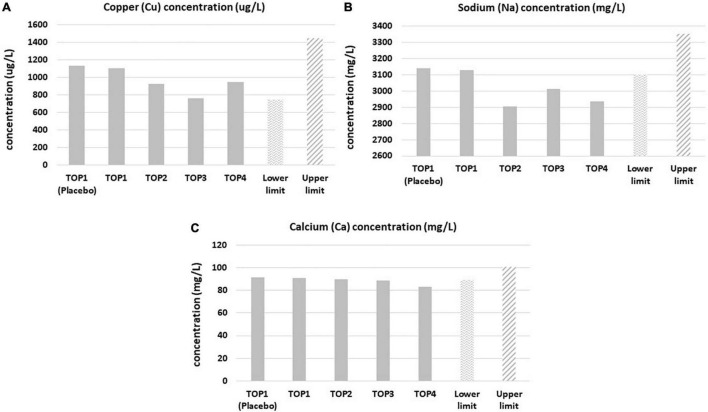
Copper **(A)**, sodium **(B)**, and calcium **(C)** blood levels in the Placebo and Verum groups during the osteoporosis TOP study course of 4 years with corresponding limits of the referent values. Statistically significant lower levels (*post-hoc*, *p* < 0.05) were observed in the third year for copper, sodium and calcium and in the fourth year for calcium and sodium in comparison with the lower referent values. TOP1-Placebo (without PMA-zeolite supplementation); TOP1-Verum, end of the first year of PMA-zeolite supplementation; TOP2-Verum 2Y, end of the second year of PMA-zeolite supplementation; TOP3-Verum 3Y, end of the third year of PMA-zeolite supplementation; TOP4-Verum 4Y, end of the fourth year of PMA-zeolite supplementation.

Mineral levels were in the referent range values, even though statistically relevant fluctuations were observed during the course of the TOP study (Table 5 and Supplementary Tables 9–13). Cu, Na, and Ca mineral levels were fluctuating below the reference range values during the course of treatment (Figure 2). For example, Cu levels were below referent values in the third year of the study, but they normalized again after completion of the fourth year of the study. Na levels remained below referent values in the second, third and fourth years of supplementation. Similarly, Ca levels were below referent values in the third and fourth years of PMA-zeolite supplementation. This may be at least partially attributed to the bone remodeling process and osteoblast-induced deposition of alkaline minerals, primarily Ca but also Na or Mg in the bone (18, 19). Thus, supplementation of these minerals may be considered as an adjuvant intervention in the long-term PMA-zeolite supplementation regimen of patients with osteoporosis.

In summary, long-term PMA-zeolite supplementation in osteoporotic patients (4 years) diminished the levels of Ca and Na and decreased the levels of metal contaminants As, Ni, and Al. Altered TOP study-Pb levels diminished over time probably as a consequence of the bone remodeling process.

### Morbus Crohn Study

A description of the study is given in the Supplementary Material (Section 2). After measurement, the standard blood parameters in subjects enrolled within the Morbus Crohn study were not affected with statistical relevance (*p* < 0.05) in any tested group, including PMA-zeolite-treated subjects. Additionally, the values for minerals or metals were also not affected with statistical relevance (*p* < 0.05) in any tested group (Supplementary Tables 5–8). The only significantly changed value was measured in patients with Morbus Crohn who were supplemented with PMA-zeolite at the end of the study, where the As levels diminished significantly in comparison with patients with Morbus Crohn without PMA-zeolite supplementation (Table 6). There were no statistically significant changes in the other measured blood parameters.

**TABLE 6:** Arsenic (As) blood concentrations analysis within the Morbus Crohn study groups.

	Arsenic (As) blood concentrations analysis within	
	the Morbus Crohn study groups (*p*-values)	
	
	Healthy volunteers	Morbus Crohn patients
Placebo	0.3505	0.8975
PMA-treated Morbus Crohn patients	0.9850	0.0499*

Comparisons were performed between the following groups: Healthy volunteers, Morbus Crohn patients, Placebo-treated healthy volunteers, and Placebo-treated Morbus Crohn patients. Statistically significant difference between the observed groups are evidenced by an asterisk (*p < 0.05).

In summary, the PMA-zeolite supplementation for 12 weeks did not significantly alter mineral and metal contaminants levels in tested subjects (healthy volunteers and Morbus Crohn patients), except for As which levels decreased significantly in PMA-zeolite supplemented patients with Morbus Crohn.

## Discussion

**FIGURE 3:**
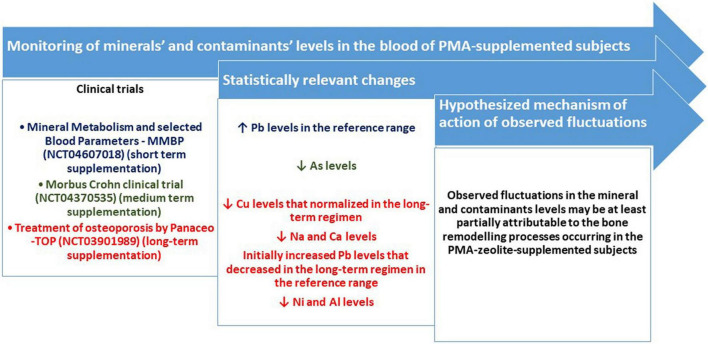
Schematic visualization of the monitoring of minerals’ and contaminants’ levels in the blood of PMA-supplemented subjects and major results listed as statistically relevant changes in particular mineral or contaminant levels (*p* < 0.05).

Clinoptilolite materials have been generally accepted as safe for *in vivo* applications if other essential requirements are met, including the quality control of the material and production process (2). This is also a natural material that might be evaluated as a promising future micro- or nano-based antioxidant devices against environmental pollutants induced-toxicities (20–22). Nevertheless, controlled clinical studies with the focus on the safety of clinoptilolite materials have not been extensively performed so far. This is probably due to the generally accepted safety of the material in animals and humans at EFSA recommended dosages (13). Still, mineral and metal homeostasis in the organism underlies all biological processes and eventual changes in this domain may be crucial in health outcomes. A tribomechanically activated clinoptilolite material has been previously studied within the OECD rules based toxicology-study by Pavelic et al. and proved as generally safe. Still, the authors did not monitor the changes in mineral and contaminant levels in tested animals (3, 4). In addition, one clinical study comprising 22 human subjects was performed previously where the treatment with activated clinoptilolite from 7 to 30 days, led to removal of toxic heavy metals from the body *via* urine (10). Another available clinical study by Zhakov (23), showed that no substantial changes in physiologically relevant trace elements or vitamins were observed even after long-term administration. Still, a comprehensive monitoring of the relevant fluctuations of mineral and contaminants levels in human subjects supplemented with controlled clinoptilolite materials, within controlled clinical trials with different supplementation regimens, were not performed so far. Herein, we accordingly, present results obtained within three controlled clinical trials (Figure 3). The clinoptilolite zeolite material used within these trials was a registered and certified medical device in the EU, the PMA-zeolite material. Within all studies, the levels of minerals and metals were monitored at the beginning of the study and at the end of PMA-zeolite supplementation (at the end of the study). The working hypothesis underlying these measurements was based on the assumption that, if this strong cation exchanger material is performing an unwanted uptake of physiologically relevant cations (minerals) in the gut and/or is hampering their absorption from the gut into the body, changes in mineral concentrations should be visible in different regimens of PMA-zeolite supplementation in the PMA-zeolite-supplemented subjects. In addition, with the premise that the material has a strong affinity for metals, as published previously, the concentrations of metals should not be increased in the blood of PMA-zeolite-supplemented subjects. The results obtained within the MMBP study showed that PMA-zeolite-supplemented volunteers had no substantial or clinically relevant alterations in biochemical and hematological parameters or physiological levels of metals, including Na and Ca, upon PMA-zeolite intake that may be attributable to PMA-zeolite. In contrast to the TOP study, Na and Ca were in the normal range and were not decreased. Moreover, decreased concentrations of the contaminants Hg and Cd after 28 days of treatment with PMA were measured in the NAÏVE group. It should be mentioned that levels Hg and As were increased above referent values for some subjects both in the NAÏVE and CHRONIC groups. This might be correlated with the Mediterranean diet and fish intake, as all subjects have reported regular sea fish intake at least once a week or more (24). Still, these values were below toxic values, and organic As accumulated from fish or other seafood bears a lower relevance for health. Indeed, inorganic As is more toxic than organic As species; organic arsenic species, such as arsenobetaine and different arsenosugars, are the most common forms in seafood (25). Interestingly, in the Morbus Crohn study, statistically decreased levels of As (*p* < 0.0499) were observed in the PMA-zeolite-supplemented patients at the end of the study (Table 6). Contrary to the MMBP study, the patients in the Morbus Crohn study were on the continental diet with a low intake of fish. Moreover, statistically higher Pb values in the MMBP studies were measured in the CHRONIC group in comparison with the NAÏVE group, with concentrations within the referent range. The elevated Pb concentrations in the PMA-zeolite-supplemented subjects within the MMBP study might be indicative of either (a) leakage of Pb from the material into the blood or (b) activation of detoxification mechanisms in the organism due to mobilization of previously accumulated Pb in the body compartments, particularly from the bones. Pb is indeed deposited in the bones and can be resorbed and released into the blood during bone remodeling, as a result of enhanced bone resorption in some pathologies (e.g., hyperthyroidism) or in certain altered states, including pregnancy, lactation, postmenopausal declines in estrogen, and aging (26). Accordingly, in the NAÏVE group, blood measurements were additionally performed 1 h after the first dose of PMA-zeolite supplementation, as this is the timeframe when uptake of contaminants from the intestine is expected to be visible in the blood (e.g., for Al or Pb) (27). However, the levels of Al and Pb (except for one subject) were not increased 1 h after the first dose of PMA-zeolite supplementation. For Al, the concentrations were not increased after 28 days of PMA-zeolite supplementation. Al levels were also normal in the CHRONIC group. It should be noted that the levels of metals in the blood and organs may also vary due to several reasons (e.g., nutrition, environmental exposure, individual status, genetics, and disease) and should thus be interpreted always by taking into account the general picture and eventual toxic symptoms (28). The observed increase of Pb levels was hypothesized to be indicative for mobilization of stored Pb from bones as a side effect of bone remodeling in PMA-zeolite-supplemented subjects. Indeed, within the osteoporosis TOP study (4 years of continuous PMA-zeolite supplementation), statistically higher levels of Pb (*p* < 0.001*) were also observed at the end of the fourth year of the clinical trial in the Verum group (TOP4) in comparison with the Placebo group. The Pb values of the Verum groups, however, remained within the reference range in the first three years (TOP1-TOP3) and started to decrease in the fourth year of application (TOP4). The Pb levels were still in the reference range, and the PMA-zeolite-supplemented subjects had improved clinical parameters indicative of bone remodeling (15). Scientific evidence supports a high affinity of clinoptilolite for Pb (29–32), and as evidenced in the herein presented studies, additional concentrations were not detectable in the blood of subjects 1 h after uptake of PMA-zeolite. Moreover, Pb is mainly accumulated in the bones (long-term storage) and is continuously remobilized as part of the physiologic remodeling of bone that accompanies the growth process or in bone remodeling conditions (33, 34). Interestingly, Pb concentrations remained statistically unaltered in the patients with Morbus Crohn within the Morbus Crohn study upon PMA-zeolite supplementation. Expectedly, Al levels were statistically significantly lower in PMA-zeolite-treated patients in comparison with Placebo within the osteoporosis TOP study, as well after the end of the fourth year of the clinical trial (TOP4) (*p* < 0.001), confirming the Al-detoxification properties of clinoptilolite materials (8). Mean mineral levels measured in the osteoporosis TOP study were in the normal reference ranges for all PMA-zeolite-supplemented groups, except for lower Cu levels after the third year in PMA-zeolite-supplemented patients, which, however, normalized again after completion of the fourth year of the study. The Na level was lower in the second, third and fourth years in PMA-zeolite-supplemented patients, which is similar to the Ca level that dropped slightly below the referent value in the third year of PMA-zeolite supplementation. However, the values of both minerals were within the reference ranges in the CHRONIC group of PMA-zeolite users (up to 8 years) of the MMBP study. In patients with osteoporosis, the reduction of Na and Ca, as building elements of the bones, is probably due to the bone modeling process observed in the osteoporosis TOP clinical trial and could thus be additionally supplemented along with PMA-zeolite intake in this group of patients.

## Conclusion

According to herein presented data on relevant mineral and contaminants levels in human subjects supplemented with a certified clinoptilolite material (PMA-zeolite) within three clinical trials with different supplementation regimens, PMA-zeolite did not increase levels of metal contaminants in the blood, which corroborates the stability of the PMA-zeolite material in the intestine upon oral intake. This is especially relevant for Pb or Al that are part of the structure of natural clinoptilolite, and their leakage from the material was not detected in the blood of the subjects who were tested. All measured metal levels were within the reference range in the short- and medium-term applications. The long-term data collection suggests a mobilization of Pb from long-term storage (e.g., bones), which may underlie the observed small increase of Pb values measured in the blood (still within reference range). The values of Pb started to decrease in the fourth year of application. All measured values of minerals and trace elements are within reference range in the short- and medium-term applications. In the long-term application over 4 years, Cu first dropped under reference range and normalized again after completion of the fourth year of application. Na and Ca dropped below the reference value in patients with osteoporosis but were within normal range in the long-term users within the MMBP study. For patients with osteoporosis and in the long-term PMA-zeolite supplementation for more than one year, its prescription may be recommended along with regular checking of the balances of the minerals Cu, Ca, and Na. Finally, the overall safety of the clinoptilolite materials, such as PMA-zeolite, along with presented safety data pointing to a detoxification potential for certain metal contaminants, may be exploited to study the usage of these natural materials as promising future micro- or nano-based antioxidant devices against environmental pollutants induced-toxicities.

## Study Limitations

### Mineral Metabolism and Selected Blood Parameters Study

The MMBP study has a low number of involved subjects not allowing for conclusive statistical conclusions. The study may be used for general assessment of the effects of PMA-zeolite on the blood concentrations of minerals and contaminants but not for other conclusions related to mineral homeostasis in the body. In addition, the study was composed of a population with typical Mediterranean habits, in particular a seafood-rich diet, which clearly influenced Hg and As levels. A genetically different population and/or a population with different lifestyle and nutritional habits may exert different results and may respond to PMA-zeolite supplementation differently.

### Morbus Crohn Study

The Morbus Crohn study has a low number of involved subjects not allowing for conclusive statistical conclusions. In particular, this is due to the drop out of six subjects. The study may be used for assessment of the general effects of PMA-zeolite on blood concentrations of minerals and contaminants but not for other conclusions related to mineral homeostasis in the body. In contrast to the MMBP study, the effect of the Mediterranean diet was reduced in the Morbus Crohn study, since all participants were from the interior of Slovenia, where eating habits are different and are based on a continental diet.

### Limitations Regarding the Generalization of the Findings

The data refer to a specially micronized natural clinoptilolite, referred to as PMA-zeolite. A generalization of the findings presented in this manuscript to other clinoptilolites is not easily accomplished without additional clinical studies, since previous findings demonstrate that the micronization technology (Panaceo Micro Activation–PMA) changes the biophysical properties of natural clinoptilolite (patent WO2018/100178A1). Therefore, further clinical studies on selected blood parameters with differently processed natural clinoptilolites should provide additional clarity.

## Data Availability Statement

The original contributions presented in the study are included in the article/Supplementary Material, further inquiries can be directed to the corresponding author.

## Ethics Statement

The studies involving human participants were reviewed and approved by the MMBP study (https://clinicaltrials.gov/, NCT04607018) was approved by the Ethical Review Committee of the General Hospital Pula, Croatia (December 17, 2015, register number: 10095/15.1). The osteoporosis TOP study (https://clinicaltrials.gov/, NCT03901989) was approved by the Ethical Committee of the University of Rijeka, Department of Biotechnology on April 3, 2014 (reference number 001-2013). Ethical approval for extension of the study to TOP3 and TOP4 was also obtained by the Polyclinic K-Center Ethical Committee (permission from February 5, 2018). The Morbus Crohn study (https://clinicaltrials.gov/, NCT04370535) was approved by the Slovenian Ethical Review Committee before the conduction (0120-203/2017-5, KME 50/04/17 and 87/06/17, date: June 21, 2017). The patients/participants provided their written informed consent to participate in this study.

## Author Contributions

SK drafted the manuscript, supervised the clinical trials experimental procedures, interpreted data results, prepared figures and tables, performed literature search, led the MMBP study, and secured the corresponding funding. JS collected samples, supervised laboratory blood analyses, and helped in clinical interpretation of obtained results in the MMBP study. DK collected samples, supervised laboratory blood analyses, and helped in clinical interpretation of obtained results in the osteoporosis TOP studies. ŽP collected samples, supervised laboratory blood analyses, and helped in clinical interpretation of obtained results in the Morbus Crohn study. MŽ performed statistical analysis of data from the osteoporosis TOP studies. LS helped in ICP-MS sample preparation and performed statistical analyses of the Morbus Crohn study. SE provided technical support. TO performed ICP-MS measurements and prepared ICP-MS results. KP performed the design of clinical trials, supervised clinical trials, secured the funding, participated in interpretation of clinical data, and performed final revision of the manuscript. All authors agreed to be accountable for all aspects of the work and approved the submitted version.

## Conflict of Interest

SK and KP were independent scientific advisors of Panaceo International GmbH, Austria. SE was employed at Panaceo International. GmbH, Austria, and involved in technical support to PMA-zeolite medical device documentation and clinical trials randomization. The remaining authors declare that the research was conducted in the absence of any commercial or financial relationships that could be construed as a potential conflict of interest.

## Publisher’s Note

All claims expressed in this article are solely those of the authors and do not necessarily represent those of their affiliated organizations, or those of the publisher, the editors and the reviewers. Any product that may be evaluated in this article, or claim that may be made by its manufacturer, is not guaranteed or endorsed by the publisher.

## Footnotes

1
https://clinicaltrials.gov/ct2/show/NCT05178719

## Abbreviations

PMA-zeolite: registered and certified clinoptilolite material
ICP-MS: inductively coupled plasma mass spectrometry
MMBP study: mineral metabolism and selected blood parameters study
TOP study: treatment of osteoporosis by Panaceo study
Pb: lead
Al: aluminum
Na: sodium
Ca: calcium
Cu: copper
As: arsenic
Ni: nickel
EFSA: European Food Safety Authority.
