# Supplementary material for: Clinical Evaluation of a Defined Zeolite-Clinoptilolite Supplementation Effect on the Selected Blood Parameters of Patients
Source: Front Med (Lausanne). 2022 May 27;9:851782. doi: 10.3389/fmed.2022.851782 (PMC9197155; doi:10.3389/fmed.2022.851782)
Supplement: Supplementary file 1 [file Table_1.DOCX]

**Supplementary material that provides additional description and results obtained within the clinical trials NCT03901989, NCT04370535, NCT04607018 presented in the main paper: Clinical evaluation of a defined Zeolite-Clinoptilolite supplementation effect on the selected blood parameters of patients by Sandra Kraljević Pavelić et al. (Correspondence: p**rof. Sandra Kraljević Pavelić,^,^ Faculty of Health Studies, University of Rijeka, Rijeka, 51000, Croatia, [sandrakp@uniri.hr](mailto:sandrakp@uniri.hr) )

**Content**

[1. Supplementary data for the clinical trial on healthy subjects (Mineral Metabolism and selected Blood Parameters, MMBP) (NCT04607018) 2](#_Toc88561070)

[1.1. Study design 2](#_Toc88561071)

[1.1.1. Subjects 2](#_Toc88561072)

[1.1.2. Treatment and time schedule 2](#_Toc88561073)

[1.2. Ethical aspects, recruitment, randomization 4](#_Toc88561074)

[1.3. Measured blood parameters 4](#_Toc88561075)

[1.3.1. Minerals K, Na, Zn. 4](#_Toc88561076)

[1.3.2. Minerals Fe, Mg, Ca, liver and renal functions: AST - Aspartate-aminotransferase, ALT - Alanine-aminotransferase, GGT -Gamma-glutamyltransferase, creatinine (CREAT) and Glomerular filtration evaluation (GF). 4](#_Toc88561077)

[1.3.3. Concentration of free Fe^3+^: MHgb – methaemoglobin. 5](#_Toc88561078)

[1.3.4. Contaminants measurements (Inductively Coupled Plasma Mass Spectrometry-ICP-MS analysis of serum and plasma samples) 5](#_Toc88561079)

[1.4. Statistical Analyses and sample size 6](#_Toc88561080)

[1.5. Results – supplementary tables S3 and S4 7](#_Toc88561081)

[2. Supplementary data for the Morbus Crohn study (NCT04370535) 9](#_Toc88561082)

[2.1. Summary of the study 9](#_Toc88561083)

[2.2. Hypothesis, goals and merits of the study 10](#_Toc88561084)

[2.3. Methods 10](#_Toc88561085)

[2.3.1. Subjects 10](#_Toc88561086)

[2.3.2. Inclusion Criteria 11](#_Toc88561087)

[2.3.3. Exclusion criteria 11](#_Toc88561088)

[2.4. Treatment and time schedule 11](#_Toc88561089)

[2.5. The measured blood parameters 12](#_Toc88561090)

[2.6. Study design 12](#_Toc88561091)

[2.6.1. Sample size and randomization 12](#_Toc88561092)

[2.6.2. Statistical analysis 13](#_Toc88561093)

[2.7. Ethical approval and study registration 13](#_Toc88561094)

[2.8. Relevant results 13](#_Toc88561095)

[3. Supplementary data for the TOP Study (NCT03901989) 19](#_Toc88561096)

# Supplementary data for the clinical trial on healthy subjects (Mineral Metabolism and selected Blood Parameters, MMBP) (NCT04607018)

## Study design

This clinical trial took place in Croatia, under supervision of the University of Rijeka at the General Hospital Pula, Croatia and the effect of the PMA-zeolite supplementation of 28 days (in accordance with the regulations of this medical device) on two groups of 15 subjects (CHRONIC and NAÏVE users) was evaluated.

### Subjects

The test population comprised 2 groups and in total 15 volunteers. **Group A (NAÏVE)** consisted of new PMA-zeolite users (7 test persons) which began to take PMA-zeolite for the first time for 28 days. All of them were healthy volunteers, males and females of a mean age of 45 years. **Group B (CHRONIC/ control)** consisted of frequent daily users (8 test persons) which already take PMA-zeolite since 28 days or even longer. They were also healthy males and females of a mean age of 56 years. Inclusion criteria for all groups were healthy volunteers (at least 18 years old), provided informed consent. Exclusion criteria (only for naïve group) were chronic disease (cancer, renal disease, neuro-degenerative, metabolic disorders, diabetic), recent vaccinations, pregnancy or breastfeeding, food supplements.

### Treatment and time schedule

All subjects received boxes containing PMA-zeolite powder for supplementation for a total of 28 days. Dosage for PMA-zeolite (100% zeolite; 1 ML – spoon for dosage = ca. 3 g zeolite), powder was as follows:

- 1 spoon of the powder stirred 2 times daily (morning and evening) in water (1 glass).
- To avoid eventual interactions with drugs, the supplementation of PMA-zeolite was suggested to be performed 1 hour after taking other drugs.

Besides the instruction for use at the beginning the trial all volunteers had to specify details about their health status/ habits in order to recognize any connection between those and the measured parameters if needed. This details comprise information about any suffering or illness (e.g.: allergy), nutritional habits, drug consumption (smoking, alcohol, medicaments or supplements) and any other relevant information (e.g.: working in metal industry). The details are summarized in the Tables S1 and S2.

**Table S1. Information of volunteers participating in the study – NAÏVE group.**

**Table S2.** **Information of volunteers participating in the study – CHRONIC group.**

The determined parameters for the mineral metabolism, the liver and kidney as well as the free Fe ^3+^ and the ICP-MS analysis of heavy metals in blood and Al were measured as follows:

1. At the beginning, at day 0 the test consisted of:
   1. Measurement of all defined parameters before any supplementation (Naïve group) and the measurement of an initial value after at least 28 days of supplementation (Chronic group) in blood and serum (Test_0, T0)
   2. Measurement after 1 hour of the supplementation of 1 spoon (ca. 3g) of PMA-zeolite (Naïve group) in blood and serum (Test_1, T1)
2. At the end, after 28 days all defined parameters were measured in blood and serum (Naïve and Chronic group). (Test_2, T2)

In summary, parameters were measured in the NAÏVE and CHRONIC groups at the beginning of the study (T0 for both groups and T1 only in the NAÏVE group) and after 28 days (T2 for both groups). Heavy metals for the chronic group were no effects attributable to PMA-zeolite were expected were measured at time-point Test_2.

## Ethical aspects, recruitment, randomization

The study was conducted according to the guidelines of the Declaration of Helsinki for Research on Human Subjects 1989 and was approved by the Ethical Review Committee of the General Hospital Pula, Croatia. This trial has been approved by the Ethical Review Committee of the General Hospital Pula, Croatia (17th December, 2015., register number: 10095/15.1). In accordance to the defined goals and hypothesis of this study no randomization took place. All included subjects were healthy volunteers selected through the University of Rijeka or the General Hospital Pula. All of the subjects provided written informed consent prior to participating in this investigation.

## Measured blood parameters

### Minerals K, Na, Zn.

**Analyses:** The method for **Na** and **K** assessment relies on indirect potentiometer analysis (Architect c4000, Abbott Diagnostics, Chicago, IL, USA). **Zn** concentrations in serum were assessed by use of spectrophotometric method by deprotonation on the analyser Roche Cobas 6000 (Roche Diagnostics, Mannheim, Germany) by use of reagent Colorimetric Method Zinc (Randox, Crumlin, UK) with cation-specific chromogen for Zn 2-(5-bromo-2-pyridylazo)-5-(N-propyl-N-sulphopropylamino)-phenol.

**Referent values/range used for analyses:** **Na**: 137 – 146 mmol/L; **K:** 3,9 – 5,1 mmol/L; **Zn:** M: 11,1−19,5 μmol/l F:10,7−17,5 μmol/l

### Minerals Fe, Mg, Ca, liver and renal functions: AST - Aspartate-aminotransferase, ALT - Alanine-aminotransferase, GGT -Gamma-glutamyltransferase, creatinine (CREAT) and Glomerular filtration evaluation (GF).

**Analyses:** Biochemical evaluation of creatinine, aspartate-aminotransferase, alanine-aminotransferase, gamma-glutamyltransferase, and Fe, Ca and Mg were done by use of biochemical analyser Architect c4000 (Abbott Diagnostics, Chicago, IL, USA) by standard spectrophotometric methods that are in accordance with suggested methods of International Federation of Clinical Chemistry and Laboratory Medicine (IFCC). The methods were used in combination with the use of verified and harmonized referent intervals suggested by Croatian chamber of medical biochemists.

Evaluation of the glomerular filtration speed was done by use of the formula from 2009 (<https://www.kidney.org/professionals/kdoqi/gfr_calculator>). Briefly, the creatine concentration is used in the formula along with declared isotope dilution mass spectrometry (IDMS) according to the American national standard for creatine (National Institute for Standards and Technology (<http://www.nist.gov/>) - NIST SRM 967, adjusted with sex and age variables of the analysed patients).

**Referent values/ range used for analyses:** **Fe:** M:11-32; F:8-30 umol/l, **Mg:** 0,65 – 1,05 mmol/L, **Ca:** 2,14 – 2,53 mmol/L**, AST:** 8-38 U/L, **ALT:** 10 - 48 U/L (lower values are not clinically relevant), **GGT:** Men: 11 – 55 U/L Woman: 9 – 35 U/L, **CREAT:** Woman: 63 – 107 µmol/L, Men: 79 – 125 µmol/L, **GF – evaluation:** of glomerular filtration G1>=90/ G2 60-80/G3a 45-59/G3b 30-40/G4 15-29/G5 <15. M: 11-32; F:8-30 µmol/l

### Concentration of free Fe^3+^: MHgb – methaemoglobin.

**Analyses:** For methaemoglobin analysis**,** complete arterial blood was collected in heparin EasyDraw Lite containers. The method relies on CO-oximetry method (Instrumentation Laboratory - IL Company, Bedford, MA, USA) by use of GEM OPLTM analyser (Instrumentation Laboratory - IL Company, Lexington, MA, USA).

**Referent values/range used for analyses:** **MHgb:** 0-1,5%; Fe M:11-32; F:8-30 umol/l

Referent values for these sets of parameters were set by Croatian chamber of medical biochemist and according to recommendation of the “National board for clinical-laboratory standards of the International federation for clinical chemistry (document NCCLS C28-P, 1992.)” where results are shown as 0.025-0.975 parts in non-parametric distribution, “A Proposal for an IUPAC-IFCC Recommendation. Syntax and Semantic Rules for Quantities and Units in Clinical Laboratory Sciences 1993: stage 1, draft 4.” and “Archiving of laboratory documentation, Journal HKMB, 8/01”.

### Contaminants measurements (Inductively Coupled Plasma Mass Spectrometry-ICP-MS analysis of serum and plasma samples)

Contaminant analyses (Pb, Cd, Hg, and As in blood and Al and Ni in serum) were carried out by using inductively coupled plasma mass spectrometry (ICP-MS, Agilent 7500cx, Agilent Technologies, Japan). Individual standard solutions (1000±7 mg/L in 4-10% HNO_3_ or 20% HCl) for the determination of heavy metals and internal standard elements, Ge, Rh, Tb and Ir, and Lu were obtained from SCP SCIENCE (SCP Science, Quebec, Canada). Particular attention was paid to precautions against external contamination and all samples were prepared and analysed in the laboratory equipped with a heating, ventilating and air conditioning (HVAC) system combined with high-efficiency particulate air (HEPA) filters. Blood and serum samples were diluted (1:70 and 1:20, respectively) before analysis in a solution containing 0.7 mM ammonia, 0.01 mM EDTA, 0.07 % Triton X-100 and 3 µg/L of internal standard (Ge, Rh, Tb and Ir) in ultrapure water (GenPure System, TKA, DE). Each sample was prepared and analysed in duplicate. Blanks, reference materials, and calibration standards were prepared in the same way as the samples and analysed accordingly. The calibration and instrument sensitivity control was performed by re-analysing selected calibration standards every 30 analyses.

Referent values/rage used for analyses: The referent values used for analysis of obtained data are set in the laboratory at the Institute of Medical Research and Occupational Health – Zagreb and are generally in line with literature data (8,9). The following upper limits were used: Lead (Pb) ≤ 50 µg/L, Mercury (Hg) <10 µg/L, Cadmium (Cd) < 1 µg/L, Arsenic (As) < 12 µg/L, Aluminium (Al) < 8 µg/L, Nickel (Ni) < 2 µg/L

## Statistical Analyses and sample size

The statistical analyses were performed after the collection of all data and at the University of Rijeka, Croatia by using the program Statistica 12 (StatSoft; p<0.05). Non-parametric analysis Mann-Whitney U and Friedman ANOVA for two independent set of data were performed for comparison of test groups: CHRONIC vs. NAÏVE, NAÏVE_0 vs. NAÏVE_1 vs. NAÏVE_2.

The sample size was based on the main question about the effect of PMA-zeolite on the mineral metabolism and selected blood parameters. The sample size of around 15 subjects is also used in the literature for clinical trials ^[[1]](#footnote-1)^

## Results – supplementary tables S3 and S4

**Table S3.** Standard blood parameters reference values within the MMBP study.

| **Blood parameter** | **Reference values** |
| --- | --- |
| **AST**  **ALT**  **GGT**  **CREAT**  **GF – evaluation: of glomerular filtration** | 8-38 U/L  10 - 48 U/L (lower values are not clinically relevant)  M: 11 – 55 U/L F: 9 – 35 U/L  F: 63 – 107 µmol/L, M: 79 – 125 µmol/L  G1>=90/ **G2** 60-80/**G3a** 45-59/**G3b** 30-40/**G4** 15-29/**G5** <15. M: 11-32; F:8-30 µmol/L |

**AST - Aspartate-aminotransferase, ALT - Alanine-aminotransferase, GGT - Gamma-glutamyltransferase, Creatinine - CREAT, Glomerular filtration evaluation -GF*

**Table S4.** Parameters for liver, kidney and Fe^3+^ (methaemoglobin) in subjects enrolled in the MMBP study. Standard biochemical laboratory blood analyses results: MHgb – methaemoglobin, creatinine, AST – Aspartate – aminotransferase, ALT – Alanine-aminotransferase, GGT – Gamma-glutamyltransferase, Glomerular filtration evaluation (GF). Beginning of the study, T0; blood sampling upon 1h of PMA-zeolite oral intake, T1; End of study upon 28 days of PMA-zeolite-supplementation, T2. * Values exceeding referent parameters are denoted in bold and red; # parameters that improved towards referent values upon PMA-zeolite-supplementation are denoted in blue and bold.

# Supplementary data for the Morbus Crohn study (NCT04370535)

## Summary of the study

Crohn´s disease (CD) is a chronic progressive destructive disease. Approximately one-fifth of adult and paediatric patients already have evidence of a structuring or penetrating intestinal complications at diagnosis. Despite decades of research, the aetiology of CD remains unknown. Disease treatments include a variety of approaches like dietary changes and immunosuppressive anti-TNFα antibodies as well as ancillary antibiotic therapy. Another interesting and promising approach in this context might be the supplementation of the certified medical device PMA-(Panaceo micro activation)-zeolite (a specific natural zeolite-clinoptilolite) due to its properties documented through many studies. In particular, the promising clinical results from a randomized controlled trial (RCT) with PMA-zeolite need to be highlighted. The strengthening/ supporting of the intestinal wall integrity in healthy subjects suffering from intestinal problems was measured in this RCT and is claimed as intended main action of the PMA-zeolite. Based on these results the application of PMA-zeolite is an interesting and promising approach in patients with uncontrolled CD.

This pilot study aims to evaluate the safety and efficacy of PMA-zeolite when used in patients with uncontrolled CD. The effect of the PMA-zeolite will be compared between a control-group (healthy volunteers) receiving either PMA-zeolite or placebo and the test-group (suffering from uncontrolled CD) and receiving either PMA-zeolite or placebo. In addition, this pilot-study should furthermore provide indications for the effect-size in order to estimate an effect for an eventual further human trial.

## Hypothesis, goals and merits of the study

The Clinical trial aims to evaluate and measure effects of the PMA-zeolite on the clinical symptoms, intestinal wall integrity and microbiota status in the gut of patients with uncontrolled CD.

Therefore, the hypothesis (H1) of this study on patients with uncontrolled CD are:

1. **Reduction of local and systemic inflammation**: an assumed positive effect of PMA-zeolite on the amelioration of the intestinal wall status/ intestinal barrier integrity and for a positive influence on the systemic and local inflammation. **(primary outcome)**;
2. **Changes (positive) of the microbiota in the gut:** an assumed positive effect of PMA-zeolite on the local intestinal status/ the microbiome (which is based on zeolite detoxification effects) **(secondary outcome)**;
3. **Positive detoxifying effect**: PMA-zeolite is assumed to remove harmful substances (contaminants) from the intestine along with release of trace elements which may positively impact microbiota status in the gut and decrease dysbiosis (decreased negative influence of pathogens on inflammation in the gut) **(tertiary outcome)**;
4. **Improvement of quality of life:** an envisaged positive systemic impact on the well-being) **(quarterly outcome)**

Goal of this intervention is, as hypothesized, an improvement of the enumerated values after an application for 12 weeks. This means that the results will help to identify how to support patients suffering from uncontrolled Crohn disease.

**The null – hypothesis (H0-Hypothesis) in detail:**

1. Supplementation has no effect on the intestinal wall integrity and therefore no effect on the systemic and local inflammation.
2. Supplementation has no effect on the microbiome/ normal bacterial flora in the gut.
3. Supplementation has no effect on the parameters of local inflammation and systemic detoxification
4. The supplementation has no impact on the quality of life

## Methods

### Subjects

The test population will comprise four groups:

| **Group A** | control group + placebo (5 test persons)* |
| --- | --- |
| **Group B** | control group + PMA-zeolite (5 test persons)* |
| **Group C** | patients with uncontrolled Crohn disease + placebo (15 patients) |
| **Group D** | patients with uncontrolled Crohn disease + PMA-zeolite (15 patients) |

**Groups A and B are healthy volunteers (see: inclusion and exclusion criteria below) and the starting-group is homogenous in age (30-60Y) and sex (male and female).*

Pre- selection and selection criteria of healthy volunteers will include absence of gastroenterology symptoms and any regularly medication prescription assessed by anamnesis. These including criteria should be confirmed by informed consent signing.

### Inclusion Criteria

- Healthy volunteers (at least 18 years old) for first two groups (A,B), homogenous starting group in age (30-60Y) and sex (m/f )
  - The health-status will be confirmed through anamnesis.
- Groups C and D will include patients with confirmed Crohn's disease that are treated with standard therapy and despite of treatment do not achieve the appropriate disease remission. Patients with the intermediate type of disease will be also included in groups C and D.
  - Diagnosis has to be confirmed with a biopsy of the intestine and histological exam.
  - Degree of disease will be assessed by Harvey-Bradshaw Index (HBI). For evaluation of quality BDQ questionnaire will be used. For the assessment of disease activity ordinary laboratory measurements will be performed (CBC, basic biochemical parameters, CRP), and calprotectin from chairs. Colonoscopy must be done in the last six months prior to inclusion of patients in the research protocol. Gastrointestinal system degree of impairment will not have influence on inclusion criteria and patients with histological intermediate type of disease will be included as well.
- Signed informed consent as per usual recommendations in vigour in the Republic of Slovenia.

### Exclusion criteria

- Signs of acute bacterial infection (fever >38°C, nausea, vomiting).
- Other chronic disease (cancer, renal disease, neuro-degenerative, metabolic disorders, diabetic).
- Pregnancy or breastfeeding
- Food supplements

## Treatment and time schedule

All subjects will receive boxes containing PMA-zeolite or placebo for supplementation for a total of 12 weeks. Dosage for 100% PMA-zeolite or placebo which is similar in appearance (either powdered product in stick pack or boxes) and was used as follows:

- 1 spoon of the powder stirred 2 times daily (morning and evening) in water (1 glass). The subjects may consume 1 spoon with the first meal of the day and 1 spoon with the last meal of the day.
- To avoid eventual interactions with drugs, the supplementation of PMA-zeolite will be suggested as supplementation 2 hours before or 1 hour after taking other drugs.
- The dosage of 1 spoon (=1 ML) is around 3 g zeolite

## The measured blood parameters

Standard parameters measurements in the blood, heavy metals in the blood and analysis of heavy metals and minerals in the serum were performed. Standards blood analysis: SR (sedimentation), K-Leukocytes, K-Erythrocytes, K-Hemoglobin, K-Hematocrit, MCV, MCH, MCHC, RDW, K-Platelets, MPV, Neutr. Segmented, Lymphocytes, Monocytes, Eosinophils, Basophils, Neutr. Segmented Lymphocytes, Monocytes (%), Eosinophils (%), Basophils (%), S-Bilirubin cel., S-AST, S-ALT, S-gamma-GT, S-Urea, S-Creatinine, oGF, S-Potassium, S-Sodium, S-Chlorine, S-Calcium, S-Iron (III), Transferrin saturation, S-UIBC, S-TIBC, S-CRP, S-Ferritin, S-Magnesium and Calprotectin. Blood contaminants: Pb, Hg, Cd, As. Minerals: Na, Ca, Mg, Fe, K, Se, Mn, Cu. All parameters were measured at 3 time-points (T0, T1 and T2).

## Study design

The pilot-study is a randomized, placebo-controlled and double blinded study. The 4 subject-groups are consisting of group A and B with healthy subjects (A and B are divided into a placebo-group and into a PMA-zeolite- group) and group C and D which are subjects suffering from CD (C and D are divided into a placebo-group and into a PMA-zeolite- group). No washout phase is planned, but if food-supplements are taken this will be protocolled. The diagnostics will be performed through the defined parameters at defined time-points.

The following questioners were used:

- Stool protocol (number of stools and description, consistence, blood presence, mucus presence)
- Food-protocol: day before measurements and in between there is to keep a protocol in order to document the food habits. The protocol will document type of food at breakfast, lunch, dinner and snacks.
- Psycho-social wellbeing and stress perception are evaluated with the “Quality of life” questionnaire (SF36)
- Degree of disease will be assessed by Harvey-Bradshaw Index (HBI). For evaluation of quality BDQ questionnaire will be used.
- Symptoms and degree will be assessed by descriptive diary: abdominal pain (according to VAS from 1-10), increased body temperature, presence of other symptoms, type of therapy and time of intake.
- Report form about adverse events for each patient

### Sample size and randomization

As the calculation of a sample size is not possible due to missing usable indications for the effect-size, a pilot-study is conducted in order to estimate an effect for an eventual further human trial. Anyhow, the sample size of around 40 subjects is adequate for clinical trials^[[2]](#footnote-2)^. The subjects will be randomized into blocks of 2 subjects (1 verum and 1 placebo in each block) whereas group A and B with the healthy subjects built a group for randomization and group C and D built a group for randomization**.** The subjects are sequentially numbered **(**[www.randomizer.org](http://www.randomizer.org)). The allocation of the powdered product/boxes will be conducted before the first application of PMA-zeolite (T1). The boxes were delivered altogether by mail.

### Statistical analysis

The statistical analyses were performed after the collection of all data and at the University of Rijeka, Croatia by using the program Statistica 12 (StatSoft; p<0.05).

## Ethical approval and study registration

The study is conducted in accordance the guidelines of the Declaration of Helsinki for Research on Human Subjects 1989 and was approved by the Slovenian Ethical Review Committee before the conduction (0120-203/2017-5, KME 50/04/17 and 87/06/17, date: 21^st^ June 2017).

## Relevant results

PMA-zeolite-treatment did not affect standard blood parameters at statistical relevance (p<0.05) in PMA-zeolite-treated subjects.

No clinically relevant changes in standard blood parameters were observed in the course of the study. Majority of standard blood values before and after the PMA-zeolite-treatment were within the reference values.

PMA-zeolite did not affect mineral or metal values at statistical relevance (p<0.05) in any PMA-zeolite-treated subject. No significant differences (p<0.05) were observed in quality of life parameters measured by use of standard questionnaires in any group comparison. Results of the contaminants’ analyses (Pb, Hg, Cd, As) in the whole blood samples are shown at the Tables S5 and S6. Significant change was observed only for As concentration in Chron’s patients after the PMA-zeolite-treatment with significance (p=0.0049). Lastly, results of heavy metals and mineral content in serum also showed no statically significant changes (p<0.05) among analysed groups (Tables S7 and S8).

PMA-zeolite-supplementation may be therefore, regarded as safe in the monitored time-frame of the study (12 weeks) in terms of effects on standard blood values and mineral and contaminants content.

**Table S5.** Results of the contaminants’ analyses (Pb, Hg, Cd, As) in whole blood samples of Placebo-treated healthy volunteers and PMA-zeolite-treated healthy volunteers. First measurement (time-point T0); measurement at the end of study (time point T1). Results are expressed as mean values ± standard deviation (SD).

| **Contaminants – blood c(µg/L)** | | | | | | | | |
| --- | --- | --- | --- | --- | --- | --- | --- | --- |
|  | **Pb T0** | **Pb T1** | **Hg T0** | **Hg T1** | **Cd T0** | **Cd T1** | **As T0** | **As T1** |
| **Placebo** **healthy volunteers** | 25.8±18.6 | 21.2±6.3 | 1.0±0.5 | 1.5±1.7 | 0.4±0.4 | 0.4±0.4 | 1.1±0.4 | 1.4±0.9 |
| **PMA-zeolite-treated healthy volunteers** | 30.9±12.0 | 32.0±13.8 | 1.3±0.7 | 1.0±0.7 | 0.2±0.1 | 0.2±0.1 | 1.7±0.5 | 1.7±0.7 |

**Table S6.** Results of the contaminants’ analyses (Pb, Hg, Cd, As) in whole blood samples of Placebo-treated Crohn patients and PMA-zeolite-treated Crohn patients. First measurement (time-point T0); measurement at the end of study (time point T1). Results are expressed as mean values ± standard deviation (SD).

|  | **Contaminants – blood c(µg/L)** | | | | | | | |
| --- | --- | --- | --- | --- | --- | --- | --- | --- |
|  | **Pb T0** | **Pb T1** | **Hg T0** | **Hg T1** | **Cd T0** | **Cd T1** | **As T0** | **As T1** |
| **placebo** | 19.4±15.7 | 17.5±12.2 | 0.9±0.9 | 0.8±0.8 | 0.5±0.3 | 0.4±0.3 | 1.2±0.8 | 1.2±0.6 |
| **PMA-zeolite** | 15.3±11.7 | 18.7±13.3 | 1.5±1.7 | 1.1±0.9 | 0.7±0.6 | 0.8±0.6 | 2.2±1.9 | **1.2±0.5*** |

** Statistically significant difference between the observed groups (p value <0.05)*

**Table S7.** Results of the contaminants and mineral analyses in the serum of Placebo-treated healthy volunteers and PMA-zeolite-treated healthy volunteers. First measurement (time-point T0); measurement at the end of study (time point T1). Results are expressed as mean values ± standard deviation (SD). No statistically relevant differences (p<0.05) were observed.

| **Contaminants and minerals – serum** | | | |
| --- | --- | --- | --- |
|  | | **Placebo-treated healthy volunteers** | **PMA-zeolite-treated healthy volunteers** |
| Ni T0 | c (µg/L) | 0.4±0.1 | 0.4±0.0 |
| Ni T1 | c (µg/L) | 0.4±0.2 | 0.4±0.2 |
| Al T0 | c (µg/L) | 3.8±0.9 | 4.4±0.1 |
| Al T1 | c (µg/L) | 4.4±0.6 | 4.5±0.5 |
| Na T0 | c (mg/L) | 3057.2±26.9 | 3089.2±0.5 |
| Na T1 | c (mg/L) | 3066.6±44.9 | 3087.4±34.0 |
| K T0 | c (mg/L) | 266.2±312.2 | 155.2±28.7 |
| K T1 | c (mg/L) | 153.7±12.5 | 159.8±7.0 |
| Mg T0 | c (mg/L) | 18.2±1.6 | 19.4±11.7 |
| Mg T1 | c (mg/L) | 18.3±1.9 | 19.6±1,0 |
| Ca T0 | c (mg/L) | 91.9±2.1 | 91.6±1.8 |
| Ca T1 | c (mg/L) | 89.7±3.1 | 90.8±2.7 |
| Se T0 | c (µg/L) | 70.9±8.5 | 65.3±2.1 |
| Se T1 | c (µg/L) | 67.1±8.7 | 63.6±7.3 |
| Zn T0 | c (µg/L) | 798.3±52.8 | 765.3±10.4 |
| Zn T1 | c (µg/L) | 763.9±88,0 | 761.8±80.4 |
| Cu T0 | c (µg/L) | 808.8±196.7 | 832.4±117.3 |
| Cu T1 | c (µg/L) | 814.2±158.4 | 826.4±160.3 |
| Fe T0 | c (µg/L) | 1125.9±488.6 | 1012.3±157.6 |
| Fe T1 | c (µg/L) | 1059.8±621.7 | 1160.1±198.4 |

**Table S8.** Results of the contaminants and mineral analyses in the serum of Placebo-treated Crohn patients and PMA-zeolite-treated Crohn patients. First measurement (time-point T0); measurement at the end of study (time point T1). Results are expressed as mean values ± standard deviation (SD). No statistically relevant differences (p<0.05) were observed.

| **Contaminants and minerals – serum** | | | |
| --- | --- | --- | --- |
|  | | **Placebo-treated Crohn patients** | **PMA-zeolite-treated Crohn patients** |
| Ni T0 | c (µg/L) | 0.4±0.1 | 0.4±0.0 |
| Ni T1 | c (µg/L) | 0.4±0.2 | 0.4±0.2 |
| Al T0 | c (µg/L) | 3.8±0.9 | 4.4±0.1 |
| Al T1 | c (µg/L) | 4.4±0.6 | 4.5±0.5 |
| Na T0 | c (mg/L) | 3057.2±26.9 | 3089.2±0.5 |
| Na T1 | c (mg/L) | 3066.6±44.9 | 3087.4±34.0 |
| K T0 | c (mg/L) | 266.2±312.2 | 155.2±28.7 |
| K T1 | c (mg/L) | 153.7±12.5 | 159.8±7,0 |
| Mg T0 | c (mg/L) | 18.2±1.6 | 19.4±11.7 |
| Mg T1 | c (mg/L) | 18.3±1.9 | 19.6±1.0 |
| Ca T0 | c (mg/L) | 91.9±2.1 | 91.6±1.8 |
| Ca T1 | c (mg/L) | 89.7±3.1 | 90.8±2.7 |
| Se T0 | c (µg/L) | 70.9±8.5 | 65.3±2.1 |
| Se T1 | c (µg/L) | 67.1±8.7 | 63.6±7.3 |
| Zn T0 | c (µg/L) | 798.3±52.8 | 765.3±10.4 |
| Zn T1 | c (µg/L) | 763.9±88,0 | 761.8±80.4 |
| Cu T0 | c (µg/L) | 808.8±196.7 | 832.4±117.3 |
| Cu T1 | c (µg/L) | 814.2±158.4 | 826.4±160.3 |
| Fe T0 | c (µg/L) | 1125.9±488.6 | 1012.3±157.6 |
| Fe T1 | c (µg/L) | 1059.8±621.7 | 1160.1±198.4 |

# Supplementary data for the TOP Study (NCT03901989)

**Table S9. Concentrations of selected metals in Verum 1Y (time point 1) and Verum 2Y (time point 2) in comparison with each other and referent values. Presented are mean values ± SD. Statistically relevant differences at p<0.05 (*).**

| **Concentration** | ***N*** | **Verum 1Y** | ***N*** | **Verum 2Y** | ***p^1^*** | ***Referent***  ***Values /*µg/L** |
| --- | --- | --- | --- | --- | --- | --- |
| **c(Pb)** **blood**/µg/L, *mean ± SD* | 29 | 36.4 ± 15.7 | 68 | 45.3 ± 17.3 | 0.376 | ≤ 50.0 |
| **c(Hg)** **blood**/µg/L, *mean ± SD* | 29 | 0.8 ± 0.8 | 69 | 1.2 ± 1.4 | 0.155 | 0-2 |
| **c(Cd)** **blood**/µg/L, *mean ± SD* | 30 | 0.8 ± 0.8 | 69 | 0.8 ± 1.0 | 0.920 | < 5 |
| **c(As)** **blood**/µg/L, *mean ± SD* | 30 | 2.8 ± 2.1 | 69 | 3.3 ± 3.8 | 0.770 | <12 |
| **c(Ni)** **serum**/µg/L, *mean ± SD* | 30 | 0.7 ± 0.2 | 69 | 0.8 ± 0.2 | 0.002* | <2 |
| **c(Al)** **serum**/µg/L, *mean ± SD* | 30 | 6.7 ± 1.9 | 69 | 5.9 ± 2.2 | 0.371 | <10 |
| **c(Mg)** / mg/L, *mean ± SD* | 30 | 18.5 ± 1.8 | 69 | 18.3 ± 1.8 | 0.238 | 17-23 |
| **c(Ca)** / mg/L, *mean ± SD* | 30 | 90.6 ± 3.2 | 68 | 89.6 ± 3.5 | 0.438 | 89-101 |
| **c(Se)** / µg/L, *mean ± SD* | 30 | 89.3 ± 15.8 | 68 | 98.0 ± 16.4 | 0.045* | 70-150 |
| **c(Fe)** / µg/L, *mean ± SD* | 27 | 1034.7 ± 248.6 | 67 | 1016.9 ± 496.9 | 0.860 | 550–1600 |
| **c(Cu)** / µg/L, *mean ± SD* | 27 | 1081.0 ± 119.6 | 68 | 942.5 ± 224.0 | 0.100 | 750-1450 |
| **c(Zn)** / µg/L, *mean ± SD* | 27 | 708.6 ± 92.8 | 67 | 667.1 ± 96.0 | 0.134 | 660-1100 |
| **c(Na)** / mg/L, *mean ± SD* | 29 | 3113.3 ± 117.2 | 67 | 2945.9 ± 153.1 | <0.001 | 3100 - 3350 |
| **c(K)** / mg/L, *mean ± SD* | 29 | 158.7 ± 17.9 | 66 | 154.5 ± 18.1 | 0.506 | 140 - 200 |

*^1^comparison of values between groups*

**Table S10. Concentrations of selected metals in Verum 2Y (time point 2) and Verum 3Y (time point 3) in comparison with each other and referent values. Presented are mean values ± SD. Statistically relevant differences at p<0.05 (*).**

|  | ***N*** | **Verum 2Y** | ***N*** | **Verum 3Y** | ***p*** | ***Referent***  ***VaLues /* µg/L** |
| --- | --- | --- | --- | --- | --- | --- |
| **c(Pb)** **blood**/µg/L | 68 | 45.3 ± 17.3 | 56 | 44.1 ± 15.8 | 0.365 | ≤ 50.0 |
| **c(Hg)** **blood**/µg/L | 69 | 1.2 ± 1.4 | 56 | 1.2 ± 1.4 | 0.836 | 0-2 |
| **c(Cd)** **blood**/µg/L | 69 | 0.8 ± 1.0 | 56 | 0.8 ± 1.0 | 0.019* | < 5 |
| **c(As)** **blood**/µg/L | 69 | 3.3 ± 3.8 | 56 | 2.2 ± 2.5 | 0.335 | <12 |
| **c(Ni)** **serum**/µg/L | 69 | 0.8 ± 0.2 | 56 | 0.6 ± 0.3 | <0.001* | <2 |
| **c(Al)** **serum**/µg/L | 69 | 5.9 ± 2.2 | 56 | 3.6 ± 1.4 | <0.001* | <10 |
| **c(Mg)** / mg/L | 69 | 18.3 ± 1.8 | 56 | 19.6 ± 1.7 | <0.001* | 17-23 |
| **c(Ca)** / mg/L | 68 | 89.6 ± 3.5 | 56 | 88.7 ± 5.2 | <0.001* | 89-101 |
| **c(Se)** / µg/L | 68 | 98.0 ± 16.4 | 56 | 83.1 ± 14.3 | <0.001* | 70-150 |
| **c(Fe)** / µg/L | 67 | 1016.9 ± 496.9 | 55 | 892.9 ± 278.3 | <0.001 | 550–1600 |
| **c(Cu)** / µg/L | 68 | 942.5 ± 224.0 | 56 | 762.3 ± 272.9 | 0.001* | 750-1450 |
| **c(Zn)** / µg/L | 67 | 667.1 ± 96.0 | 56 | 688.1 ± 97.3 | 0.304 | 660-1100 |
| **c(Na)** / mg/L | 67 | 2945.9 ± 153.1 | 56 | 3011.6 ± 120.5 | 0.010 | 3100 - 3350 |
| **c(K)** / mg/L | 66 | 154.5 ± 18.1 | 56 | 154.5 ± 14.8 | 0.149 | 140 - 200 |

*^1^t-test for dependent samples*

**Table S11. Concentrations of selected metals in Verum 3Y (time point 3) and Verum 4Y (time point 4) in comparison with each other and referent values. Presented are mean values ± SD. Statistically relevant differences at p<0.05 (*).**

|  | ***N*** | **Verum 3Y** | ***N*** | **Verum 4Y** | ***P^1^*** | ***Referent***  ***Values /* µg/L** |
| --- | --- | --- | --- | --- | --- | --- |
| **c(Pb)** **blood**/µg/L | 56 | 44.1 ± 15.8 | 57 | 38.9 ± 15.7 | <0.001* | ≤ 50.0 |
| **c(Hg)** **blood**/µg/L | 56 | 1.2 ± 1.4 | 58 | 1.3 ± 1.6 | 0.695 | 0-2 |
| **c(Cd)** **blood**/µg/L | 56 | 0.8 ± 1.0 | 58 | 0.7 ± 1.1 | 0.336 | < 5 |
| **c(As)** **blood**/µg/L | 56 | 2.2 ± 2.5 | 58 | 2.6 ± 4.5 | 0.741 | <12 |
| **c(Ni)** **serum**/µg/L | 56 | 0.6 ± 0.3 | 58 | 0.3 ± 0.1 | <0.001* | <2 |
| **c(Al)** **serum**/µg/L | 56 | 3.6 ± 1.4 | 58 | 3.3 ± 1.0 | 0.289 | <10 |
| **c(Mg)** / mg/L | 56 | 19.6 ± 1.7 | 58 | 20.5 ± 1.9 | <0.001* | 17-23 |
| **c(Ca)** / mg/L | 56 | 88.7 ± 5.2 | 58 | 83.3 ± 5.4 | <0.001* | 89-101 |
| **c(Se)** / µg/L | 56 | 83.1 ± 14.3 | 58 | 86.0 ± 17.9 | 0.100 | 70-150 |
| **c(Fe)** / µg/L | 55 | 892.9 ± 278.3 | 58 | 918.9 ± 259.3 | 0.506 | 550–1600 |
| **c(Cu)** / µg/L | 56 | 762.3 ± 272.9 | 58 | 949.3 ± 173.2 | <0.001* | 750-1450 |
| **c(Zn)** / µg/L | 56 | 688.1 ± 97.3 | 58 | 723.9 ± 93.5 | 0.024* | 660-1100 |
| **c(Na)** / mg/L | 56 | 3011.6 ± 120.5 | 58 | 2936.5 ± 169.4 | 0.008* | 3100 - 3350 |
| **c(K)** / mg/L | 56 | 154.5 ± 14.8 | 58 | 146.9 ± 16.2 | 0.001* | 140 - 200 |

*^1^t-test for dependent sampLes*

**Table S12. Concentrations of selected metals in Placebo (time point 1) and Verum 3Y (time point 3) in comparison with each other and referent values. Presented are mean values ± SD. Statistically relevant differences at p<0.05 (*).**

|  | ***N*** | **Placebo** | ***N*** | **Verum 3Y** | ***p1*** | ***Referent***  ***Values /* µg/L** |
| --- | --- | --- | --- | --- | --- | --- |
| **c(Pb)** **blood**/µg/L | 29 | 23.7 ± 11.2 | 56 | 44.1 ± 15.8 | <0.001* | ≤ 50.0 |
| **c(Hg)** **blood**/µg/L | 29 | 0.8 ± 0.8 | 56 | 1.2 ± 1.4 | 0.159 | 0-2 |
| **c(Cd)** **blood**/µg/L | 29 | 1.5 ± 1.2 | 56 | 0.8 ± 1.0 | 0.054 | < 5 |
| **c(As)** **blood**/µg/L | 29 | 2.4 ± 1.3 | 56 | 2.2 ± 2.5 | 0.688 | <12 |
| **c(Ni)** **serum**/µg/L | 30 | 0.6 ± 0.2 | 56 | 0.6 ± 0.3 | 0.999 | <2 |
| **c(Al)** **serum**/µg/L | 30 | 5.5 ± 1.1 | 56 | 3.6 ± 1.4 | 0.013* | <10 |
| **c(Mg)** / mg/L | 30 | 19.3 ± 1. 4 | 56 | 19.6 ± 1.7 | 0.989 | 17-23 |
| **c(Ca)** / mg/L | 30 | 91.3 ± 4.2 | 56 | 88.7 ± 5.2 | <0.001* | 89-101 |
| **c(Se)** / µg/L | 30 | 92.3 ± 17.5 | 56 | 83.1 ± 14.3 | 0.416 | 70-150 |
| **c(Fe)** / µg/L | 30 | 965.8 ± 246.7 | 55 | 892.9 ± 278.3 | 0.022* | 550–1600 |
| **c(Cu)** / µg/L | 30 | 1134.4 ± 167.0 | 56 | 762.3 ± 272.9 | 0.011* | 750-1450 |
| **c(Zn)** / µg/L | 30 | 702.5 ± 96.6 | 56 | 688.1 ± 97.3 | 0.494 | 660-1100 |
| **c(Na)** / mg/L | 30 | 3140.1 ± 85.6 | 56 | 3011.6 ± 120.5 | <0.001* | 3100 - 3350 |
| **c(K)** / mg/L | 30 | 166.0 ± 14.5 | 56 | 154.5 ± 14.8 | 0.518 | 140 - 200 |

**Table S13. Concentrations of selected metals in Placebo (time point 1) and Verum 4Y (time point 4) in comparison with each other and referent values. Presented are mean values ± SD. Statistically relevant differences at p<0.05 (*).**

|  | ***N*** | **Placebo** | ***N*** | **Verum 4Y** | ***p^1^*** | ***Referent***  ***VaLues /* µg/L** |
| --- | --- | --- | --- | --- | --- | --- |
| **c(Pb)** **blood**/µg/L | 29 | 23.7 ± 11.2 | 57 | 38.9 ± 15.7 | <0.001 | ≤ 50.0 |
| **c(Hg)** **blood**/µg/L | 29 | 0.8 ± 0.8 | 58 | 1.3 ± 1.6 | 0.016 | 0-2 |
| **c(Cd)** **blood**/µg/L | 29 | 1.5 ± 1.2 | 58 | 0.7 ± 1.1 | 0.026 | < 5 |
| **c(As)** **blood**/µg/L | 29 | 2.4 ± 1.3 | 58 | 2.6 ± 4.5 | 0.816 | <12 |
| **c(Ni)** **serum**/µg/L | 30 | 0.6 ± 0.2 | 58 | 0.3 ± 0.1 | <0.001 | <2 |
| **c(Al)** **serum**/µg/L | 30 | 5.5 ± 1.1 | 58 | 3.3 ± 1.0 | <0.001 | <10 |
| **c(Mg)** / mg/L | 30 | 19.3 ± 1. 4 | 58 | 20.5 ± 1.9 | 0.003 | 17-23 |
| **c(Ca)** / mg/L | 30 | 91.3 ± 4.2 | 58 | 83.3 ± 5.4 | <0.001 | 89-101 |
| **c(Se)** / µg/L | 30 | 92.3 ± 17.5 | 58 | 86.0 ± 17.9 | 0.123 | 70-150 |
| **c(Fe)** / µg/L | 30 | 965.8 ± 246.7 | 58 | 918.9 ± 259.3 | 0.421 | 550–1600 |
| **c(Cu)** / µg/L | 30 | 1134.4 ± 167.0 | 58 | 949.3 ± 173.2 | <0.001 | 750-1450 |
| **c(Zn)** / µg/L | 30 | 702.5 ± 96.6 | 58 | 723.9 ± 93.5 | 0.322 | 660-1100 |
| **c(Na)** / mg/L | 30 | 3140.1 ± 85.6 | 58 | 2936.5 ± 169.4 | <0.001 | 3100 - 3350 |
| **c(K)** / mg/L | 30 | 166.0 ± 14.5 | 58 | 146.9 ± 16.2 | <0.001 | 140 - 200 |

1. The Lancet (2009). "Phase 0 trials: a platform for drug development?". Lancet 374 (9685): 176. [↑](#footnote-ref-1)
2. The Lancet (2009). "Phase 0 trials: a platform for drug development?". Lancet 374 (9685): 176. [↑](#footnote-ref-2)
